# Evaluation of the Community-Based Hypertension Management Programs in China

**DOI:** 10.3389/fpubh.2022.896603

**Published:** 2022-05-31

**Authors:** Guang Hao, Zuo Chen, Xin Wang, Linfeng Zhang, Yuting Kang, Congyi Zheng, Lu Chen, Zengwu Wang, Runlin Gao

**Affiliations:** ^1^Department of Public Health and Preventive Medicine, School of Medicine, Jinan University, Guangzhou, China; ^2^State Key Laboratory of Cardiovascular Disease, Division of Prevention and Community Health, National Center for Cardiovascular Disease, National Clinical Research Center of Cardiovascular Disease, Fuwai Hospital, Peking Union Medical College & Chinese Academy of Medical Sciences, Beijing, China; ^3^Department of Cardiology, Fuwai Hospital, Peking Union Medical College & Chinese Academy of Medical Sciences, Beijing, China

**Keywords:** hypertension, community-based program, control, China, public health

## Abstract

**Purpose:**

The National Essential Public Health Services Package (NEPHSP), a set of community-based hypertension management programs, was launched by the Chinese government in 2009. However, the data are limited for the comprehensive evaluation of NEPHSP on hypertension management. This study was to estimate the effect of NEPHSP on hypertension control nationwide in China.

**Methods:**

Data were from China Hypertension Survey (CHS). The participants (*n* = 119,412) aged ≥35 years with hypertension were included in the analysis. Further, a subset of 64,188 diagnosed hypertensive patients were analyzed to evaluate the effect of NEPHSP by comparing the ones covered and not covered by NEPHSP. Blood pressure (BP) was measured by trained staff using a validated digital portable monitor in local communities or clinics.

**Results:**

Among adults aged ≥35 years with hypertension, the coverage of NEPHSP was 25.6% and increased with age. The coverage was significantly higher in women than in men (*P* < 0.001). Among the 64, 188 diagnosed hypertensive patients, compared to the control group (not covered by NEPHSP), the mean systolic and diastolic BPs were 2 mmHg and 1.6 mmHg lower in NEPHSP group, respectively. The rate of treatment for hypertension was significantly higher in NEPHSP group than the control group (93.0% vs. 81.4%, *P* < 0.001), and the rate of BP control was also significantly higher in NEPHSP group than the control group (35.9% vs. 29.6%, *P* < 0.001). Furthermore, similar trends were found in rural and urban, as well as in men and women.

**Conclusions:**

Our results showed that NEPHSP is effective in improving hypertension treatment and control in hypertensive patients in China. However, the coverage of NEPHSP was still low.

## Introduction

Hypertension is the leading modifiable risk factor for cardiovascular (CV) disease, which is a major cause of mortality and morbidity in China and worldwide (1, 2). In China, the prevalence of hypertension has reached 23.2% in adults (3). Although safe and effective antihypertensive medications have been available for decades, treatment and control of hypertension remain low in China. Moreover, only 46.9% hypertensive patients were aware of their condition, 40.7% were treated with antihypertensive medications, and 15.3% had controlled blood pressure (BP) (3). The burden of hypertension and CV disease in China is expected to continue increasing along with rapid urbanization, economic growth, and aging of the population (2, 4, 5).

To address current health challenges, including obesity, diabetes, and other non-communicable diseases, and to reduce the disparities for the Chinese population in accessing essential public health services, the Chinese government issued the National Essential Public Health Services Package (NEPHSP) in 2009 (6, 7). The benefit of NEPHSP included physical examination, health education, chronic disease screening, management, etc. For hypertension, the services of NEPHSP include establishing health records, screening and following-up, and routine physical examinations of identified hypertension (8). A recent study showed that coverage of NEPHSP program was associated with increases of 7.9% in BP control, 10.3% in medication, and 10.5% in BP monitoring among adults aged ≥45 years. This study also showed that NEPHSP program helped to equalize the geographic disparities in access to health services such as BP monitoring (7). However, data are still limited for a comprehensive evaluation of NEPHSP on hypertension control in Chinese adults. This study used the China Hypertension Survey (CHS) data to examine the progress and impact of NEPHSP on hypertension control nationwide in China.

## Methods

The CHS study design was published elsewhere (9). Briefly, a stratified, multistage random sampling method was used to obtain a nationally representative sample of the Chinese population. A total of 487,349 participants aged ≥15 years were recruited, with a response rate of 66.4%. After excluding 7,507 participants due to missing information on major risk factors, 479,842 were eligible and completed the survey. The participants (*n* = 119,412) who were aged ≥35 years with hypertension were included in the analysis. Further, a total of 64, 188 hypertensive patients who were aware of having hypertension were included to evaluate the effect of NEPHSP (33,946 patients covered by NEPHSP were considered as the intervention group, and 30,242 patients not covered by NEPHSP were considered as the control group) (Supplementary Figure S1). The written informed consent was signed by each participant. The Ethics Committee of Fuwai Hospital (Beijing, China) approved the study (Approved number: 2012-402).

A comprehensive operational manual was developed to ensure standardization and high quality of data. All study investigators completed a training program that oriented them both to the aims of the study and the specific tools and methodologies employed. The questionnaire was filled and checked for completeness on site. All data were checked for inconsistencies in the entries and outlying values (9). Demographic characteristics and social-economic factors were collected using a standardized questionnaire. Bodyweight with light clothes was measured using an OMRON body fat and weight measurement device (V-body HBF-371, OMRON, Kyoto, Japan). Height was measured without shoes (to the nearest 0.5 cm). Body mass index (BMI) was calculated as weight divided by the square of height (kg/m^2^). After the participant was sitting at rest for 5 min, BP was measured three times on the right arm positioned at heart level using the OMRON HBP-1300 Professional Portable Blood Pressure Monitor (OMRON, Kyoto, Japan). The accuracy of the Omron HBP-1300 had been verified in our previous study (10).

According to 2010 Chinese guidelines for the management of hypertension (11), hypertension was defined as systolic blood pressure (SBP) ≥140 mm Hg, and/or diastolic blood pressure (DBP) ≥ 90 mm Hg, and/or use of antihypertensive medicine within 2 weeks. Awareness of hypertension was defined as self-report of any previous diagnosis of hypertension by a doctor, treatment as self-reported use of antihypertensive medication within 2 weeks, control as SBP <140 mmHg and DBP <90 mm Hg. Coverage of NEPHSP was defined as the number of hypertensive patients covered by NEPHSP divided by the number of hypertensive patients.

Overweight and obese were defined as BMI between 24.0 and 27.9 kg/m^2^ and 28.0 kg/m^2^ or above, respectively (12). Participants who consumed at least one serving of alcoholic beverage per week in the past month were defined as drinkers. Participants who smoked at least 20 packets of cigarettes in their lifetime and currently smoke cigarettes were defined as current smokers; participants who smoked at least 20 packets of cigarettes in their lifetime, and quit smoking for at least 1 month were defined as former smokers, and other participants were defined as never smokers.

Antihypertensive drugs were classified as diuretics, β-blockers, α-β-blockers, calcium channel blockers, angiotensin-converting enzyme inhibitors (ACEIs), angiotensin II receptor blockers (ARBs), centrally acting drugs, vasodilators, and traditional Chinese medicine. Single-pill combinations (SPCs), which normally contain ≥2 active ingredients, were separated into their generic components (3).

### Statistical Analysis

Survey weights were calculated according to the 2010 China population census data and the complex sampling scheme including oversampling for specific age subgroups, non-response, and other demographic between the sample (13).

Variables were summarized using means with 95% confidence intervals (CI) for continuous data; using frequencies, percentages, or proportions for categorical data. Two-tailed Student *t*-tests with the Wilcoxon rank-test when necessary and *Chi*-squared tests were used to compare continuous and categorical variables, respectively. Logistic regression, applied with “svy: logit,” was used to test the differences in hypertension treatment and control between the intervention and control group after adjusting for age, sex, BMI, ethnicity, education attainment, smoking status, consumption of alcohol, family history of hypertension, and region (rural/urban). *P* < 0.05 was the threshold for statistical significance. Statistical analyses were conducted with Stata 12.1 (STATA Corp., TX, USA).

## Results

**Figure 1:**
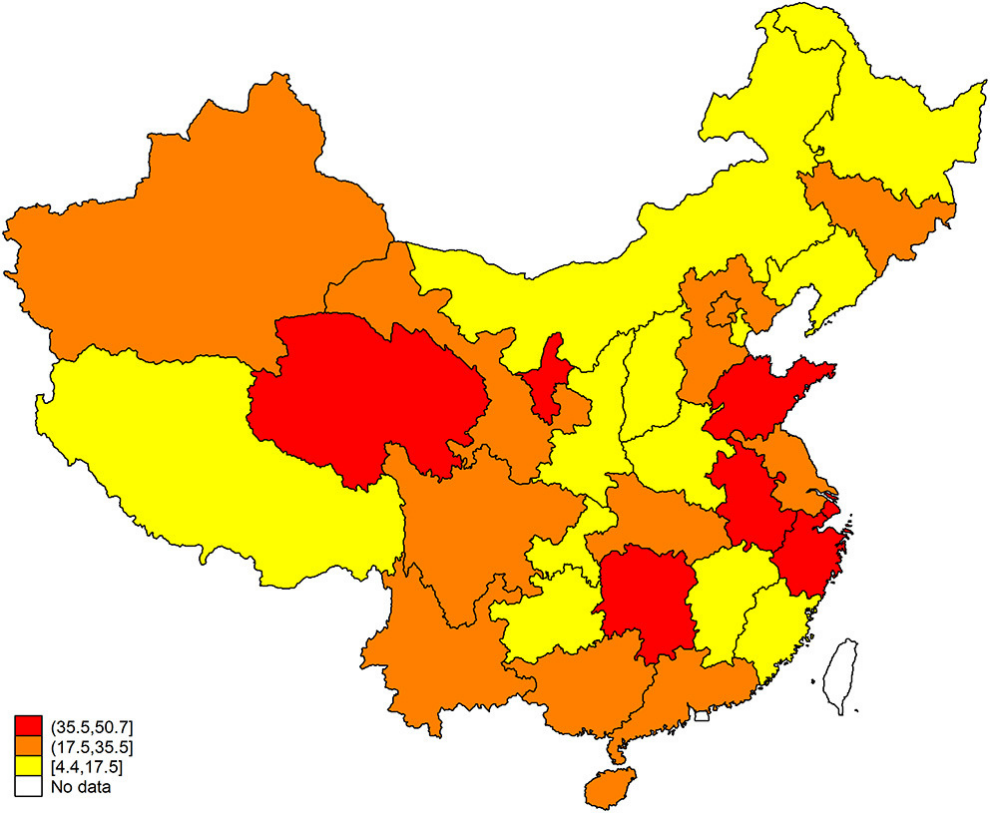
Coverage of the National Essential Public Health Services Package by Province.

A total of 119,412 participants with hypertension aged ≥ 35 years (52.8% women and 53.1% rural) completed the survey and were included in the analysis. Overall, the coverage of NEPHSP was 25.6% and increased with age (Table 1). The coverage of NEPHSP was significantly higher in women than men (*P* < 0.001), while the coverage of NEPHSP between urban and rural populations was similar (*P* = 0.361). The coverage of NEPHSP varied considerably among provinces (Figure 1). Three provinces or province-level municipalities, Shandong, Zhejiang, and Shanghai, ranked in the top 3 for coverage of NEPHSP at 50.7, 46.9, and 41.1%, respectively; whereby, Xizang had the lowest coverage of NEPHSP (4.4%) (Supplementary Table S1).

**Table 1:** Coverage of the National Essential Public Health Services Package by Region and Sex, and Province.

**Province**	**Region**		**Sex**		**Total**	***P*-value for region**	***P*-value for sex**
	**Urban**	**Rural**	**Men**	**Women**			
*N*	56,037	63,375	56,888	62,524	119,412		
**Age**							
35–44	13.4 (9.6–18.4)	11.8 (8.8–15.6)	11.0 (8.5–14.1)	14.5 (11.8–17.7)	12.3 (9.8–15.2)	0.553	0.002
45–54	24.1 (18.9–30.0)	22.0 (16.4–28.7)	20.4 (16.8–24.5)	25.3 (20.6–30.6)	22.7 (18.7–27.3)	0.608	<0.001
55–64	31.1 (25.4–37.4)	26.7 (20.5–34.1)	25.8 (21.6–30.6)	30.8 (25.9–36.2)	28.3 (23.8–33.4)	0.334	<0.001
65–74	35.4 (28.2–43.4)	31.7 (24.1–40.3)	31.5 (26.0–37.5)	34.3 (28.7–40.5)	33.0 (27.5–39.0)	0.492	0.001
≥75	37.4 (29.7–45.7)	30.8 (22.5–40.6)	32.1 (26.2–38.6)	33.8 (27.4–40.9)	33.1 (26.9–39.9)	0.282	0.09
Overall	28.2 (22.5–34.7)	24.1 (18.3–31.2)	22.9 (18.9–27.4)	28.5 (23.6–33.8)	25.6 (21.2–30.5)	0.361	<0.001

*All values were weighted to represent the total population of Chinese aged 18 years or older based on Chinese census 2010*.

A total of 64, 188 hypertensive patients who were aware of having hypertension were included to evaluate the effect of NEPHSP. Compared to the control group, the mean SBP and DBP were 2 mmHg and 1.6 mmHg lower in NEPHSP group, respectively (Table 2). The mean age and percentage of women were higher in NEPHSP group, while the percentages of participants with higher education levels, smokers, and alcohol drinkers were lower in NEPHSP group (*P* < 0.05).

**Table 2:** Difference between the intervention and control groups.

**Characteristics**	**Control**	**Intervention**	***P*-value**
*n* (%)	30,242 (47.1)	33,946 (52.9)	
Mean age, yr	58.6 (58–59.2)	61 (60.6–61.5)	<0.001
Sex (Female, %)	50 (48.3–51.8)	53.8 (52.5–55)	<0.001
Mean BMI, kg/m^2^	26 (25.5–26.4)	25.7 (25.3–26)	0.137
<18.5	1.6 (1.1–2.1)	2.1 (1.6–2.8)	
18.5–23.9	28.7 (25.4–32.3)	31.3 (27.7–35.1)	0.143
24.0–27.9	41.8 (40.5–43.1)	41.6 (40.3–43)	
≥28.0	27.9 (23.8–32.4)	25 (21.7–28.6)	
Mean SBP—mmHg	149.1 (147.6–150.6)	147.1 (145.4–148.8)	0.009
Mean DBP—mmHg	85.6 (84.8–86.4)	84.2 (83.3–85)	0.001
Ethnicity **(**Han, %)	93.1 (89–95.7)	90.7 (80.9–95.8)	0.359
Educational attainment, %			
Elementary school	52 (48.1–55.8)	58.1 (52.7–63.2)	
Elementary middle school	43.6 (40.3–46.9)	38.1 (33.9–42.5)	0.021
High school or above	4.4 (3.6–5.5)	3.8 (2.7–5.4)	
**Smoking status, %**			
Non-smokers	69.9 (67.5–72.2)	74.3 (72.1–76.4)	
Past smokers	6.8 (5.6–8.2)	6 (5.1–7)	0.001
Current smokers	23.3 (21.7–25)	19.8 (18.2–21.5)	
Consumption of alcohol, %	19.5 (17.5–21.5)	16.5 (14.3–19)	0.018
Family history of hypertension, %	46 (41.3–50.7)	43.8 (39.5–48.2)	0.353
Rural, %	62.6 (49–74.5)	61.4 (48.1–73.3)	0.881

*BMI, body mass index; SBP, systolic blood pressure; DBP, diastolic blood pressure*.

*Data are represented as value (95% CI)*.

*All values were weighted to represent the total population of Chinese aged 18 years or older based on Chinese census 2010*.

The overall prescribing patterns of antihypertensive drugs in intervention and control groups were similar except for Traditional Chinese medications and single-pill combinations of ACEIs and diuretics, which were more commonly used in the intervention group (*P* < 0.001) (Table 3).

**Table 3:** Antihypertensive medicine use in participants with hypertension.

**Variables**	**Control**	**Intervention**	***P*-value**
**Drug Type for monotherapy** ^†^			
β-blockers	4.2 (3.3–5.4)	4.1 (2.9–5.8)	0.910
Diuretics	6.6 (4.6–9.3)	7.6 (5.7–10.2)	0.423
CCBs	42.0 (35.9–48.3)	41.7 (35.6–48)	0.925
ACEIs	14.6 (12.7–16.7)	17.3 (13.7–21.5)	0.169
ARBs	9.1 (6.3–12.8)	11.5 (6.8–18.7)	0.284
α-β-blockers	0.1 (0.0–0.1)	0.1 (0.0–0.5)	0.633
CADs	9.3 (6.4–13.3)	9.6 (5.2–17.0)	0.916
Vasodilators	0.4 (0.1–1.7)	0.2 (0.1–0.5)	0.417
TCMs	5.5 (3.9–7.6)	3 (2.2–4.1)	<0.001
**Single-pill combinations**			
ACEIs and Diuretics	0.1 (0.0–0.3)	0.5 (0.2–1.3)	<0.001
ARBs and Diuretics	0.5 (0.2–1.0)	0.3 (0.2–0.5)	0.108
CADs/Vasodilators/Diuretics^‡^	17.4 (14.3–21.0)	15.7 (12.4–19.6)	0.157
ARB and CCBs	0.0 (0.0–0.2)	0.1 (0.0–0.1)	0.781
Combination of TCMs	0.7 (0.1–2.9)	0.6 (0.2–1.4)	0.633
**Number of drugs for combination therapy** ^**§**^			
1	83.8 (79.0–87.7)	87.7 (82.1–91.8)	0.547
2	3.7 (2.5–5.3)	3.1 (2.4–3.9)	
≥3	11.9 (8.4–16.4)	9 (5.3–14.9)	

*ARBs, angiotensin II receptor antagonists; ACEIs, angiotensin-converting-enzyme inhibitors; CCBs, calcium-channel blockers; TCMs, Traditional Chinese medications; CADs, Centrally acting drugs*.

*^†^ The classes of drugs were used as monotherapy, not pooled with the ingredients of the single-pill combinations*.

*^‡^ Considered Chinese traditional antihypertensive combinations, which normally contain reserpine, hydralazine, and diuretics*.

*^§^ Single-pill combinations (SPCs), which normally contain ≥2 active ingredients, were separated into their generic components*.

**Figure 2:**
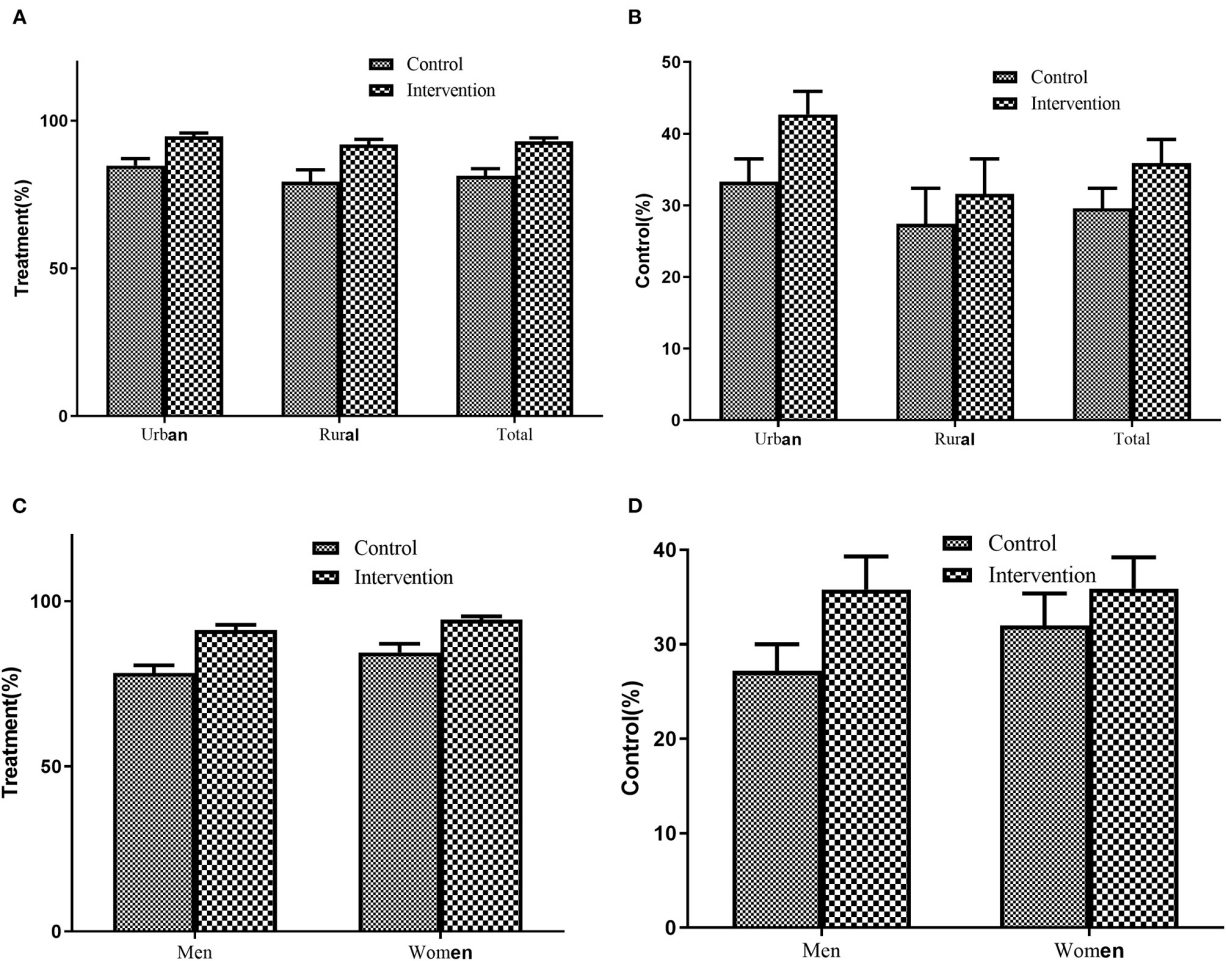
Treatment and Control of Hypertension between intervention and control group. **(A)** Treatment by region, **(B)** Control by region, **(C)** Treatment by sex, **(D)** Treatment by sex. Adjusting for age, sex, BMI, ethnicity, education attainment, smoking status, consumption of alcohol, family history of hypertension, and region (rural/urban).

After adjusting for other covariates, the treatment rate of hypertension was significantly higher in the intervention group than the control group (93.0% vs. 81.4%, *P* < 0.001), and the BP control rate was also significantly higher in the intervention group than the control group (35.9% vs. 29.6%, *P* < 0.001). Furthermore, similar trends were found in both rural and urban, as well as in men and women (Figure 2).

## Discussion

This large survey from a national representative sample showed that only 25.6% of hypertensive patients aged ≥ 35 years in China were covered by NEPHSP, and the coverage was significantly higher in women than men, similar in urban and rural populations. Compared to the control group, the mean SBP and DBP were 2 mmHg and 1.6 mmHg lower in NEPHSP group, respectively. Furthermore, the treatment and control of hypertension were significantly higher in the intervention group than in the control group.

Studies show that even a 1-mm Hg decrease in SBP could prevent substantial numbers of CV events, including heart failure, coronary heart disease, and stroke (14). Framingham Heart Study reported that a 2-mm Hg population-wide DBP reduction was associated with a 17% decrease in the risk of hypertension and a 6% reduction in coronary heart disease (15). In NEPHSP group, the treatment, and control of hypertension were significantly higher, and the mean SBP and DBP had a 2 mmHg and 1.6 mmHg decrease, which can be translated to a significant reduction in CV diseases. The Chinese government provided a subsidy of 15 yuan ($2.14) per capita for primary care providers to deliver NEPHSP since 2009. From 2011 to 2013, increased public funding ($4.61) had been invested to expand NEPHSP services including annual physical examination for all residents aged ≥65 years, regular health check-ups, and follow-up services for patients with hypertension or type 2 diabetes aged ≥35 years (7). We roughly estimated the cost of NEPHSP and saving from only CV disease events prevented by NEPHSP according to the data from the Felodipine Event Reduction (FEVER) study and China Health Statistics Yearbook (16, 17). The results indicated that if all hypertensive patients aged ≥35 years in China were covered by NEPHSP, the cost would be 0.55 million US dollars/year, and the saving would be 0.37 million US dollars/year. Considering NEPHSP covers multiple health services, NEPHSP is very likely expected to be cost-effective, although future health economic analysis is required.

The previous study indicated that high-risk strategies have a major impact on CV disease in the population (18). Our results showed that only 25.6% of hypertensive patients aged ≥ 35 years in China were covered by NEPHSP. This result is in line with the previous study (7), which reported that only 8.1% of the middle-aged and older population took the EPHS-covered physical examination between 2011 and 2013, suggesting a great potential if NEPHSP scales up to a higher coverage. Therefore, national-wide health education, increase primary care providers and budget should be considered (19–21). There is a significant geographical diversity for coverage of NEPHSP. Shandong, Zhejiang, and Shanghai with relatively higher gross domestic products in China (22) ranked in the top 3. These results suggest that more attention should be paid to specific geographical regions.

Hypertension is a condition of long duration and slow progression that is largely preventable through the improvement of their unhealthy lifestyles, including smoking, unhealthy diet, physical inactivity, and alcohol consumption (23). A growing body of evidence supports the feasibility and benefits of implementing community-based programs for the prevention of CV disease (24, 25). Research shows that increasing the awareness of hypertension is one of the most effective ways to control BP and reduce related CV diseases (26, 27). Increasing the awareness of hypertension helps people increase their opportunity for receiving high-quality care, and helps them to lead healthy lifestyles (26). Consistent with previous results, we also found that the smoking and drinking rate was lower in hypertensive patients covered by NEPHSP.

One of the major strengths is that our study sample represented all the target population of NEPHSP and it represented the overall hypertensive population in China. There are several limitations in this study. The first limitation is that our data on NEPHSP were based on self-report, which may be subject to recall bias. Second, hypertension was diagnosed based on only 3 times BP measurements at one visit. Third, the low response rate may produce a non-response bias. Finally, more studies with randomized design will be needed to confirm our findings.

## Conclusions

Our results showed that NEPHSP is efficient in hypertension treatment and control in hypertensive patients aged ≥ 35 years in China. However, the coverage of NEPHSP was still lower. These results suggest that NEPHSP has the potential for hypertension management in China and other developing countries, thus, it should be extended.

## Data Availability Statement

The datasets presented in this article are not readily available because rights of use were only granted to the authors. Requests to access the datasets should be directed to wangzengwu@foxmail.com.

## Ethics Statement

The studies involving human participants were reviewed and approved by Ethics Committee of Fuwai Hospital. The patients/participants provided their written informed consent to participate in this study.

## Author Contributions

ZW and RG contributed to the conceptualization of the study. GH performed the statistical analysis, interpreted the results, and wrote the first draft of the manuscript. ZC, XW, LZ, YK, CZ, and LC critically reviewed the manuscript. All authors contributed to the article and approved the submitted version.

## Funding

This study was supported by the China National Science & Technology Pillar Program (2011BAI11B01) and the National Health and Family Planning Commission, China (201402002).

## Conflict of Interest

OMRON Corporation, Kyoto, Japan supported Blood Pressure Monitor (HBP-1300) and body fat and weight measurement device (V-body HBF-371); Henan Huanan Medical Science & Technology Co., Ltd, China supported Digital ECG device (GY-5000); and Microlife, Taibei, China supported for Automated ABI device (WatchBP Office device). And, this study received funding from BUCHANG PHARMA, Xian, China, Pfizer China, and Essen Technology (Beijing) Company Limited. The funder was not involved in the study design, collection, analysis, interpretation of data, the writing of this article or the decision to submit it for publication. The authors declare that the research was conducted in the absence of any commercial or financial relationships that could be construed as a potential conflict of interest.

## Publisher's Note

All claims expressed in this article are solely those of the authors and do not necessarily represent those of their affiliated organizations, or those of the publisher, the editors and the reviewers. Any product that may be evaluated in this article, or claim that may be made by its manufacturer, is not guaranteed or endorsed by the publisher.
