# Supplementary material for: Evaluation of the Community-Based Hypertension Management Programs in China
Source: Front Public Health. 2022 May 31;10:896603. doi: 10.3389/fpubh.2022.896603 (PMC9194386; doi:10.3389/fpubh.2022.896603)
Supplement: Supplementary file 1 [file Data_Sheet_1.pdf]

Supplemental Table 1. Sampling population and coverage of the National Essential Public Health Services Package by region and sex, and province (sort descending).

Province	N	Region		Sex		Total, %	*P* value for region	*P* value for sex
		Urban, %	Rural, %	Men, %	Women, %			
Shandong	12,134	55.2	49.0	46.3	55.3	50.7	<0.001	<0.001
Zhejiang	12,167	44.7	48.0	41.5	53.2	46.9	0.032	<0.001
Shanghai	5,263	42.5	19.0	38.9	43.5	41.1	<0.001	0.382
Anhui	11,849	51.3	35.2	39.6	40.8	40.2	<0.001	0.732
Hunan	13,052	50.9	37.4	35.1	44.3	39.7	<0.001	0.002
Ningxia	12,557	25.9	47.1	32.4	41.4	36.9	<0.001	<0.001
Qinghai	9,499	38.4	35.2	36.2	35.9	36.0	0.57	0.883
Xinjiang	4,829	39.1	32.8	32.6	38.7	35.5	<0.001	0.001
Beijing	8,207	36.1	11.7	29.8	40.0	34.9	<0.001	<0.001
Gansu	10,180	24.3	39.1	31.4	37.0	34.0	<0.001	0.013
Yunnan	10,131	27.1	33.1	27.8	36.7	32.0	<0.001	<0.001
Jilin	9,771	37.9	25.7	24.4	35.6	29.9	<0.001	<0.001
Hubei	13,242	30.0	24.4	24.3	30.0	26.9	<0.001	<0.001
Hebei	11,683	22.3	26.4	22.2	29.0	25.8	0.02	<0.001
Guangdong	12,409	32.3	14.4	22.3	28.0	25.0	<0.001	<0.001
Sichuan	13,012	28.0	23.9	22.3	27.6	24.8	0.159	0.045

Guangxi	11,459	17.8	23.8	21.4	22.9	22.1	<0.001	0.804
Hainan	9,743	35.7	2.0	21.5	17.8	19.8	<0.001	0.355
Jiangsu	12,455	21.2	17.0	15.7	21.4	18.4	<0.001	<0.001
Fujian	9,825	24.0	15.0	15.3	19.9	17.5	<0.001	0.005
Shanxi	8,348	9.9	19.5	16.2	17.4	16.8	<0.001	0.667
Tianjin	8,609	13.1	36.3	16.0	17.2	16.6	0.956	<0.001
Jiangxi	11,773	22.2	14.1	14.9	17.4	16.1	<0.001	0.291
Chongqing	9,628	17.9	14.0	13.4	18.3	15.8	0.002	0.006
Shaanxi	10,100	21.9	10.6	13.8	16.0	14.9	<0.001	0.044
Heilongjiang	10,371	11.3	14.5	11.2	16.0	13.3	<0.001	<0.001
Liaoning	8,826	6.2	20.1	9.6	16.7	13.0	<0.001	<0.001
Guizhou	9,542	23.8	8.8	10.4	12.9	11.6	<0.001	0.194
Inner Mongolia	10,830	16.8	7.4	7.3	14.8	10.8	0.001	<0.001
Henan	15,359	6.2	7.2	6.4	7.7	7.0	0.114	0.082
Xizang	3,556	6.4	4.1	3.7	5.2	4.4	0.117	0.132

Data are represented as sample participant number, weighted prevalence. All values were weighted to represent the total population of Chinese aged 18 years or older based on Chinese census 2010.

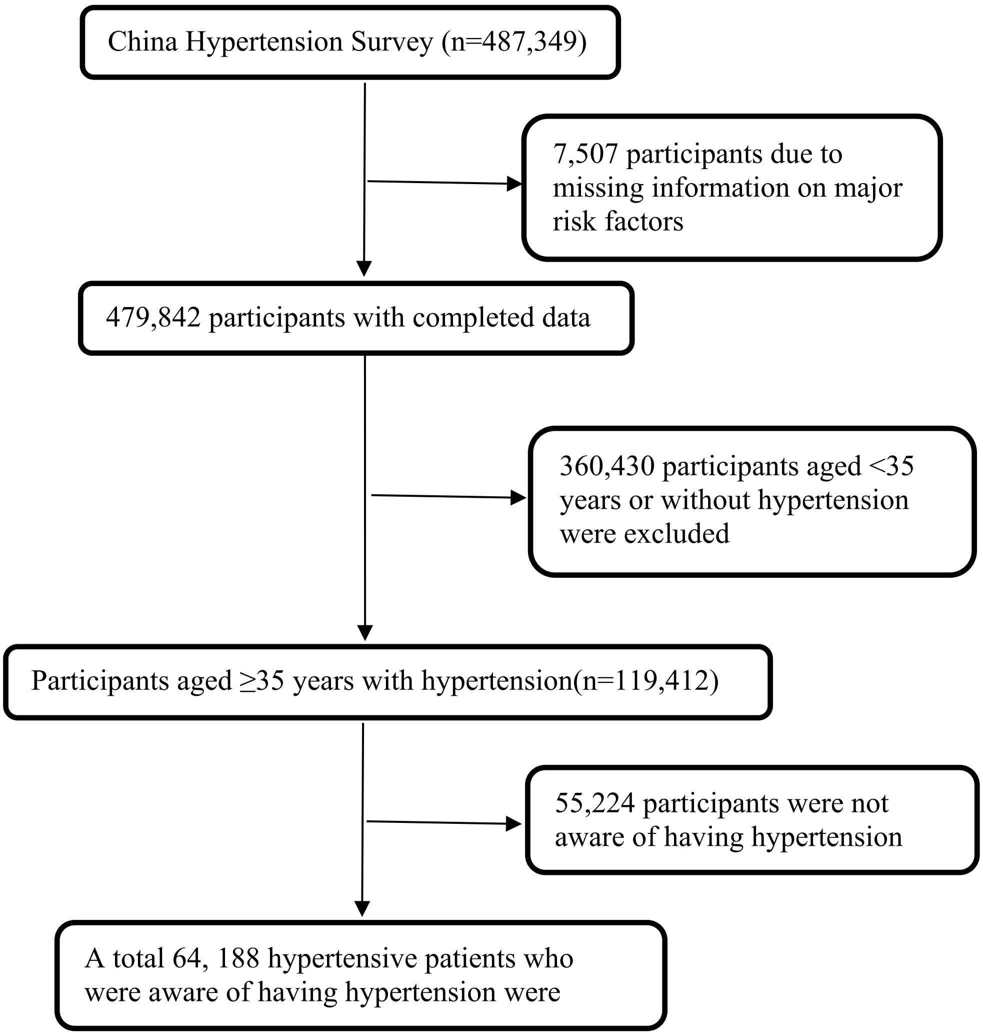

Supplemental Figure 1. Flowchart of the participants' inclusion
